# Comparison of the Protective Effects of Individual Components of Particulated* trans*-Sialidase (PTCTS), PTC and TS, against High Cholesterol Diet-Induced Atherosclerosis in Rabbits

**DOI:** 10.1155/2017/7212985

**Published:** 2017-02-27

**Authors:** Shérrira M. Garavelo, Maria de Lourdes Higuchi, Jaqueline J. Pereira, Marcia M. Reis, Joyce T. Kawakami, Renata N. Ikegami, Suely A. P. Palomino, Nilsa S. Y. Wadt, Abdelali Agouni

**Affiliations:** ^1^Laboratory of Cardiac Pathology, Heart Institute, School of Medicine, University of São Paulo, São Paulo, SP, Brazil; ^2^Pharmaceutical Sciences Section, College of Pharmacy, Qatar University, P.O. Box 2713, Doha, Qatar

## Abstract

Previous studies showed the presence of* Mycoplasma pneumoniae* (*M. pneumoniae*) and membrane-shed microparticles (MPs) in vulnerable atherosclerotic plaques. H&S Science and Biotechnology developed PTCTS, composed by natural particles from medicinal plants (PTC) combined with* trans*-Sialidase (TS), to combat MPs and* Mycoplasma pneumoniae*. Our aim was to determine the effects of the different components of PTCTS in a rabbit model of atherosclerosis. Rabbits were fed with high cholesterol diet for 12 weeks and treated during the last 6 weeks with either vehicle, PTC, TS, or PTCTS. Lipid profile and quantification of MPs positive for* Mycoplasma pneumoniae *and oxidized LDL antigens were carried out. Aortas and organs were then histologically analyzed. PTCTS reduced circulating MPs positive for* Mycoplasma pneumoniae* and oxidized LDL antigens, reduced the plaque area in the abdominal aorta, and caused positive remodeling of the ascendant aorta. PTC caused positive remodeling and reduced plaque area in the abdominal aorta; however, TS had a lipid lowering effect. PTCTS components combined were more effective against atherosclerosis than individual components. Our data reinforce the infectious theory of atherosclerosis and underscore the potential role of circulating MPs. Therefore, the removal of* Mycoplasma*-derived MPs could be a new therapeutic approach in the treatment of atherosclerosis.

## 1. Introduction

Human atherosclerosis is a chronic systemic process, characterized by the presence of arterial wall thickening due to the accumulation of lipids, endothelial dysfunction, and inflammatory and fibroproliferative responses, resulting in the formation of atherosclerotic plaques, vascular proliferation, and extracellular matrix changes [1].

When levels of LDL rise in systemic circulation, they bind to proteoglycans and increase their retention time in the arterial intima, settling on it, where they are oxidized by free radicals, triggering the inflammation that damages the endothelial barrier function, increasing thus vascular permeability [2].

Previous studies from our laboratory have shown that, in human atherosclerosis,* Mycoplasma pneumoniae (M. pneumoniae)* was present within the atherosclerotic plaques, with greater quantities in vulnerable plaques, which broke and led to acute myocardial infarction, when associated with* Chlamydophila pneumoniae* [3].* Mycoplasmas* are among the few prokaryotes that can grow symbiotically with other microorganisms, strongly interacting with mammalian host cells for long periods [4].

When exposed to stress conditions,* Mycoplasmas *can release microparticles (MPs) containing proteins and genetic material from the originating cells as a response to such stress, thus allowing their adaptation to various environments [5, 6].

MPs are small vesicles of 100 nm to 1 *μ*m in diameter, coated in membrane and released during cell activation or apoptosis. They harbor membrane proteins of the parent cell, which allows for their characterization according to their cellular origin [7, 8]. The effects of MPs may vary between procoagulant and proinflammatory properties, induction of endothelial dysfunction by changes in nitric oxide (NO) levels and in the metabolic pathway of prostacyclin, decrease in expression of NO synthase (NOS), stimulation of cytokines release such as interleukin-8 (IL-8), IL-1*β*, IL-6, and tumor necrosis factor-*α* (TNF-*α*), and the expression of cell adhesion molecules [8, 9], among other actions, fundamental to the formation and development of atheromatous plaque [10].

To prevent and treat atherosclerosis, H&S Science and Biotechnology developed a compound that was reported to fight MPs, PTCTS (particulated* trans*-Sialidase), registered in the US, INCI, under the name PTCTS (number 5-11-2013-1386). The PTCTS compound consists of natural organic particles (PTC) obtained from medicinal plants associated with* trans*-Sialidase enzyme (TS). Methods of obtaining and preparation are protected by patents granted in the United States (number 7,914,781), United Kingdom (number EP1296554B1), Canada (number CA2383850C), and Brazil (number PI0101648-8).

Higuchi [11] proposed that the use of TS could lead to the regression of the atherosclerotic process by eliminating* Mycoplasmas* through its capacity of reallocating sialic acids, a family of amino sugars, which are involved in cell-to-cell interactions and signaling functions in hematopoietic, immune, and nervous systems [12]. It is known that the sialic acids act as binders for pathogen receptors, particularly to siglecs (sialic acid-binding immunoglobulin like lectins) [13]; however, they are also important nutrients for the maintenance of* Mycoplasmas* [14]. Therefore, the relocation of sialic acids from the environment close to the bacteria to the surrounding siglecs would allow them to act as their ligands, a way of eliminating such bacteria. Santos et al. [15] have injected the PTCTS compound intramuscularly to rabbits fed high cholesterol diet to induce atherosclerosis and could regress the atherosclerotic process and reduce total cholesterol and its fractions to normal serum levels [15]; however, the underlying mechanisms and the relative contribution of PTC and TS components to the observed effects are yet to be investigated.

Therefore, the present project was designed to further understand how the protective effects of PTCTS compound are mediated and to decipher the relative contribution of its individual components, PTC and TS, to the observed effects against atherosclerosis in rabbits. In this study, we specifically assessed the effects of PTCTS, PTC, and TS on the regression of atheromatous plaques and the elimination of MPs associated with* M. pneumoniae* and oxidized low density lipoprotein (oxLDL) antigens from the serum of atherosclerotic rabbits. We have also evaluated the potential toxicity of these compounds in the kidneys, liver, and spleen from these rabbits.

## 2. Materials and Methods

### 2.1. Study Design

Male and female white New Zealand rabbits from the Central Animal Laboratory of the Faculty of Medicine (University of Sao Paulo, USP) of approximately 60 days of age and weighing 3.0 ± 0.5 kg were used in the present study. Animals were placed in stainless steel cages appropriate to the species and maintained with specific food for the species and water ad libitum, with light and dark intervals every 14/10 hours.

The sample size was calculated according to Student's distribution method described by Dunnett [16]. The following formula was used:

(1) $$\begin{array}{c} t=\frac{\overset{-}{X}-\mu }{s/\sqrt{n}}. \end{array}$$

Based on previous similar experimental studies from our group [15], we have obtained sample standard deviation (*s*) and the used value for *t* was set at 2.447 for a 95% confidence interval of a two-tailed analysis and 6 degrees of freedom.

Rabbits included in the study were randomly divided into 5 groups as follows:

1. * Normal diet group*: negative control group (CNeg, *n* = 6) which received 400 *μ*L of water/day instead of the treatment
2. * High cholesterol diet group*: positive control group (CPos, *n* = 6) which received 400 *μ*L of water/day instead of the treatment
3. * High cholesterol diet treated with organic particles group*: (PTC, *n* = 6) where animals were treated with natural organic particles (300 *μ*L/day) during the last six weeks of high cholesterol diet
4. The natural organic particles were obtained through a mixture of the medicinal plants extracts (*Ginkgo biloba, Allium sativum, Zingiber officinale*, and* Dendrobium moschatum*), ethanol, and water, according to the protocol described by Sousa et al. [17]. This mixture was diluted in thermal water 1 : 10,000 for the treatment administration
5. * High cholesterol diet treated with trans-Sialidase group*: (TS, *n* = 5) where animals were treated with the enzyme* trans*-Sialidase (1.62 × 10^−7^ mg/mL), 100 *μ*L/day, during the last six weeks of high cholesterol diet
6. * High cholesterol diet treated with PTCTS group*: (PTCTS, *n* = 6) where animals were treated with PTCTS (400 *μ*L/day) during the last six weeks of high cholesterol diet

Data obtained from the group of animals fed with normal diet were used as reference values for healthy animals, while data from positive control group, which received high cholesterol diet for 12 weeks, without treatment, were used as reference values for diseased animals.

Animals in all groups were maintained for 12 weeks and then sacrificed at the Central Animal Laboratory of the Faculty of Medicine, University of São Paulo, where the samples were harvested for further study.

All procedures involving experimentation on animals were performed in accordance with* Guide for the Care and Use of Laboratory Animals* (1996, published by National Academy Press, 2101 Constitution Ave. NW, Washington, DC 20055, USA). Animal research protocol was approved by the local ethical review committee of the University of São Paulo.

### 2.2. Diet and Treatment Preparation

For the preparation of the hypercholesterolemic diet, it was enriched with 1% cholesterol by spraying a solution containing powder cholesterol (Sigma-Aldrich, Missouri, USA), ethyl ether (Labsynth, Diadema, Brazil), and absolute ethanol (Labsynth, Diadema, Brazil) on usual commercial feed in the ratio of 1 g of cholesterol to each 100 g of feed.

Cholesterol was dissolved by adding 100 mL of ethyl ether and 50 mL absolute ethanol/g under constant stirring. The solution was mixed with the usual feed and left in exhaust hood for complete evaporation of the solvents for 24 hours.

### 2.3. Blood Collection and Biochemical Analyses

At the beginning of the experiment, prior to the start of the high cholesterol diet, blood samples were collected from the marginal ear vein of each animal for biochemical analysis of basal levels of total cholesterol (TC) and fractions [HDL and non-HDL (n-HDL) cholesterol] and triglycerides (TG). Similar procedure was performed immediately before the start of treatment with PCT, TS, or PTCTS and at the end of the experiment following the sacrifice of the animals. Samples obtained were named basal, pretreatment, and posttreatment, respectively.

Biochemical analyses of total cholesterol and its fractions were performed at the Laboratory of Clinical Analysis, while histology and immunofluorescence analyses were performed at the Cardiac Pathology Laboratory, both at the Heart Institute of the Faculty of Medicine, University of São Paulo (InCor-HCFMUSP).

### 2.4. Sacrifice of the Animals

The animals were sacrificed by first administering intramuscularly an anesthetic (ketamine, 25–50 mg/kg associated with xylazine 2–5 mg/kg) in addition to barbiturate (thiopental) to deepen the anesthetic action. Following this, animals were injected with 3 mL of potassium chloride by intracardiac route. Animals were immediately subjected to necropsy.

### 2.5. Histological Analyses of Aorta and Organs

Aorta was dissected from the monoblock of organs, sectioned at its root and iliac bifurcation. Cross sections along the aortic root containing approximately 0.5 cm extension from the ascending portion of the artery, 2 mm above the bronchial branches and 2 mm below the renal arteries, were made, and these fragments were then immediately embedded in paraffin for histological analysis.

Then, paraffin blocks were sectioned with a microtome into sections of 5 *μ*m of thickness. Sections were then stained with hematoxylin and eosin (H&E) and scanned by ScanScope CS System® (Aperio Technology, Inc., CA, USA) with an objective Olympus UPlanSAPO 20x attached to the scanner that generated image files “.svs” that were then analyzed by Aperio ImageScope View software (Aperio Technologies, Inc., California, USA).

By means of the software aid, we measured at the ascending portion of aorta the external elastic lamina perimeter (PE); the internal elastic lamina perimeter (PI); the area of the middle layer (AM); potential lumen of the vessel (LP), vessel lumen without the plaque; the real lumen of the vessel (LR), vessel lumen with the plaque; the area of atheromatous plaque (AP); and the percentage of vessel obstruction (% Obs).

In the thoracic and abdominal portions, we evaluated the total length of the fragment (Et), the length of the atheromatous plaque base (Ep), and its total area (AP).

During the sacrifice, samples of liver, spleen, and kidneys were fixed in 10% buffered formalin, pH 7.4, for 24 hours, which were processed for embedding in paraffin. The paraffin blocks were serially 5 um sectioned, stained with H&E, scanned through ScanScope CS System (Aperio Technology, Inc., CA, USA), and finally analyzed qualitatively in Aperio ImageScope View software (Aperio Technologies, Inc., CA, USA). The analysis of sections from these organs evaluated the integrity of tissues and cells, the presence of lesions, and cellular changes.

### 2.6. Immunofluorescence

Immunofluorescence technique was used to quantify the numbers of MPs positive for* M. pneumoniae* and oxLDL antigens. This analysis was performed in the serum of animals from the different stages of the protocol (i.e., at baseline, before the start of treatment, and at the end of the treatment).

To separate MPs, total serum solution was mixed with H medium (D-mannitol, sucrose, HEPES, and BSA) and then centrifuged and thus separated into two phases: pellet and supernatant. Both fractions were analyzed fresh. Immunofluorescence analysis was performed on the supernatant where MPs are present.

The serum supernatant from animals in each group was incubated with primary antibody against oxLDL (clone 2C7OSF10 prepared and donated by Dr. Dulcineia Abdalla, Faculty of Pharmaceutical Sciences, USP) or primary antibody against* M. pneumoniae *(clone B748M, Abcam, Cambridge, MA, USA).

For detection, aliquots labeled with oxLDL antibody were incubated with donkey anti-mouse secondary antibody (Alexa Fluor 488 IgG H + L) (Life Technologies, Carlsbad, CA, USA), while aliquots labeled with antibody against* M. pneumoniae* were incubated with donkey anti-mouse secondary antibody (Alexa Fluor 555, IgG H + L) (Life Technologies, Carlsbad, CA, USA).

Images of immune-stained slides with their fluorophores were captured in fluorescent microscope EVOS FL Cell Imaging System (Advanced Microscopy Group (AMG), Thermo Fisher Scientific, Waltham, Massachusetts, USA) using an objective of magnification ×20 and saved in JPG format to be quantified with the IrfanView (Irfan Skiljan, Vienna, Austria) software. The numbers of MPs were then quantified in two blind fields (4 pictures from each) from each sample, with magnification ×20.

### 2.7. Statistical Analyses

The quantitative variables obtained were expressed as mean ± SD. Statistical analysis was performed using SigmaStat for Windows, version 3.5.

The differences between CNeg × CPos were calculated using Student's *t*-test. Paired *t*-test was applied to compare data obtained from animals of the same groups at different collection times (pretreatment versus posttreatment) in serum.

For comparison of serum analysis between groups, we used the difference between the values of the pretreatment and posttreatment sampling (Δ*T*) using ANOVA test in order to verify if differences were induced by the treatment.

In the group treated with PTCTS, results obtained were compared with the untreated group (CPos) by Student's *t*-test.

Later, PTCTS and CPOs data were compared to the other groups by ANOVA (CPos × PTCTS × other treatments). When ANOVA showed significant differences, the comparison between groups was performed using the post hoc Bonferroni test for data that passed the normality test and the Dunn test for analyses that did not pass the test, always setting the CPos as control.

In all analyses, we considered *P* < 0.05 as statistically significant.

## 3. Results

### 3.1. Lipid Profile

As shown in Table 1, the biochemical analysis of samples revealed that all groups with high cholesterol diet had levels of TC and fractions, and TG changed between baseline and pretreatment stages.

**Figure 1:**
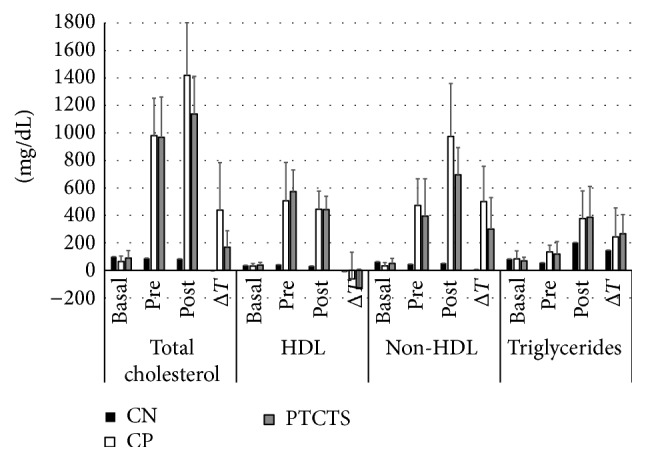
Total cholesterol, HDL cholesterol, non-HDL cholesterol, and triglyceride circulating levels (mg/dL) at baseline (basal), pretreatment (pre), and posttreatment (post) collection and Δ*T* in the serum of negative control (CN), positive control (CP), and animals treated with PTCTS. Data are expressed as mean ± SD. *N* = 6 in each group.

CPos group showed a significant increase between pretreatment and posttreatment regarding TC (*P* = 0.03), n-HDL cholesterol (*P* = 0.005), and triglycerides (*P* = 0.04). When compared to CNeg group, CPos increased significantly the TC (*P* = 0.009) and n-HDL cholesterol levels (*P* = 0.002); however, no differences were observed for the other variables (HDL, *P* = 0.82; TG, *P* = 0.09) (Figure 1).

Biochemical analysis of serum from animals in the PTCTS group showed a significant increase in TC (*P* = 0.02), n-HDL (*P* = 0.02), and triglycerides (*P* = 0.005) values and a modest decrease in the levels of HDL (*P* = 0.07) when we compared the pretreatment versus posttreatment samples. When comparing the Δ*T* difference between values of lipid profile with CPos, it was noted that they did not show any statistically significant differences (TC, *P* = 0.1; HDL, *P* = 0.49; n-HDL, *P* = 0.18; TG, *P* = 0.82).

The PTC group showed no reducing effect over lipid profile when we have compared pretreatment versus posttreatment phases (TC, *P* = 0.2; HDL, *P* = 0.42; n-HDL, *P* = 0.11; TG, *P* = 0.14) or even when Δ*T* differences were compared to CPos and PTCTS groups (TC, *P* = 0.4; HDL, *P* = 0.59; n-HDL, *P* = 0.62; TG, *P* = 0.95).

The TS group showed a lowering effect over HDL cholesterol (*P* = 0.03) and increased, although not significantly, n-HDL cholesterol (*P* = 0.08) between pretreatment and posttreatment stages, with no significant changes in the other variables (TC, *P* = 0.95; TG, *P* = 0.12). When comparing the Δ*T* differences to CPos and PTCTS groups, TS group showed a reduction in TC levels (*P* = 0.05) with no other differences (HDL, *P* = 0.32; n-HDL, *P* = 0.15; TG, *P* = 0.89).

### 3.2. Histology of the Aorta

**Figure 2:**
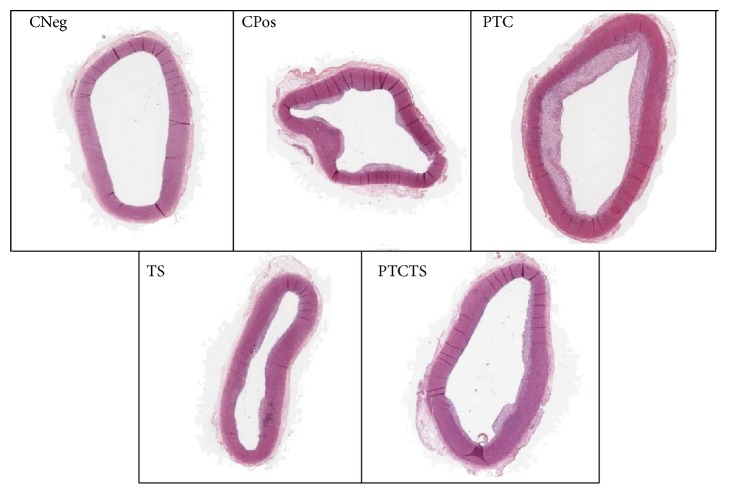
Representative images of histological analysis (H&E staining) of the ascending portion aortas of rabbits from each group. Negative control (CNeg), positive control (CPos), and animals treated with PTC, TS, or PTCTS.

The analysis of the ascending aortas of animals from CPos group showed no difference compared to CNeg group in relation to the size of the vessel (PE, *P* = 0.99; PI, *P* = 0.77; LP, *P* = 0.42; AM, *P* = 0.73); however, they had larger plaque area (*P* = 0.003), increased % Obs of the vessel (*P* = 0.004), and decreased LR (*P* = 0.04), as shown in Table 2. Animals of CPos group presented alterations in the middle layer areas, suggestive of calcification. These changes were observed only in this group (Figure 2). The analysis of the thoracic portion (Table 3) showed that both groups, CNeg and CPos, had similar Et (*P* = 0.73); however, CPos rabbits showed higher Ep (*P* = 0.004) and AP (*P* = 0.002). Similar results were observed in the abdominal aorta (Et, *P* = 0.8; Ep, *P* = 0.002; AP, *P* = 0.002).

**Figure 3:**
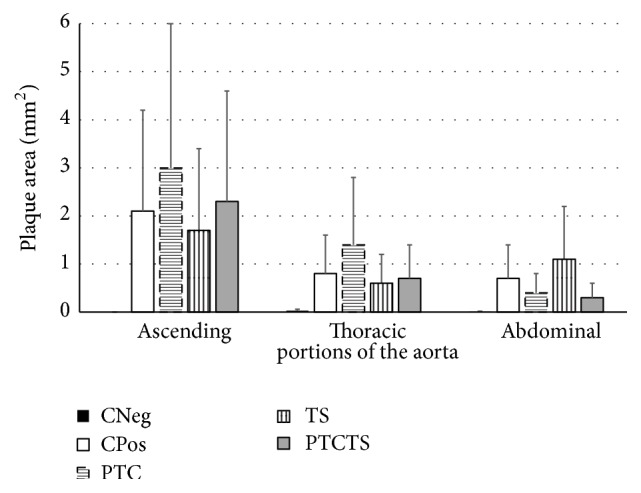
Plaque area in the ascending, thoracic, and abdominal aortas from each group. Data are expressed as mean ± SD. *N* = 5-6 in each group. Negative control (CNeg), positive control (CPos), and animals treated with PTC, TS, or PTCTS.

Animals treated with PTCTS showed a significant increase of PE (*P* = 0.01), PI (*P* = 0.04), LP (*P* = 0.01), LR (*P* = 0.04), and AM (*P* = 0.01) when compared to CPos group, with no differences in the other variables (AP, *P* = 0.41; % Obs, *P* = 0.69). The thoracic aorta of animals from the PTCTS group showed no difference in the other variables compared to CPos (Et, *P* = 0.31; Ep, *P* = 0.68; AP, *P* = 0.82). However, the abdominal aorta showed lower plaque length and plaque area in the group treated with PTCTS (CPos, Ep, 4.07 ± 2.54; AP, 0.74 ± 0.25 versus PTCTS, Ep, 3.11 ± 2,53; AP, 0.32 ± 0.29) although not significantly (Et, *P* = 0.87; Ep, *P* = 0.55; AP, *P* = 0.25) (Figure 3).

The treatment of rabbits with PTC influenced the vessel structure, by increasing the PE (*P* = 0.03), PI (*P* = 0.03), and LR (*P* = 0.03) values compared to CPos and PTCTS groups (Table 2), while the other variables did not differ (LR, *P* = 0.06; AP, *P* = 0.52; AM, *P* = 0.06; % Obs, *P* = 0.86). No histological differences were found in the thoracic (Et, *P* = 0.45; Ep, *P* = 0.44; AP, *P* = 0.33) or in the abdominal portions of the aorta (Et, *P* = 0.99; Ep, *P* = 0.55; AP, *P* = 0.67) in comparison to CPos and PTCTS groups (Table 3).

Rabbits treated with TS showed no differences in the vessel structure at histological analysis of the ascending (PE, *P* = 0.39; PI, *P* = 0.07; LP, *P* = 0.51; LR, *P* = 0.13; AP, *P* = 0.63; AM, *P* = 0.09; % Obs, *P* = 0.89), thoracic (Et, *P* = 0.61; Ep, *P* = 0.91; AP, *P* = 0.88), or abdominal (Et, *P* = 0.43; Ep, *P* = 0.6; AP, *P* = 0.19) portions of aorta when compared to CPos and PTCTS groups (Tables 2 and 3).

### 3.3. Immunofluorescence

**Figure 4:**
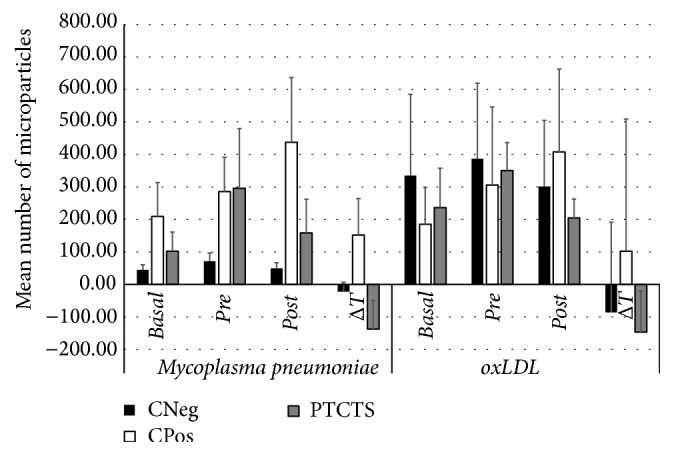
Number of circulating microparticles (MPs) associated with antigens of* Mycoplasma pneumoniae* and oxLDL in animal plasma at baseline (basal), pretreatment (pre), and posttreatment (post) collection and Δ*T* in the serum of negative control (CNeg), positive control (CPos), and animals treated with PTCTS. Data are expressed as mean ± SD. *N* = 6 in each group.

The assessment of MPs positive for* M. pneumoniae* and oxLDL antigens in serum showed that even though animals were randomly distributed to the experimental groups, animals from CPos and TS groups already had high levels of MPs positive for* M. pneumoniae *antigens (Table 4), while CNeg and TS groups had increased numbers of MPs positive for oxLDL antigens compared to other groups (Table 5) at the baseline. Therefore, we evaluated the difference between the pretreatment and posttreatment phases (Δ*T*) to determine whether treatments influenced the number of these MPs (Figure 4).

By doing so, it was possible to note a significant increase in the number of both* M. pneumoniae* and oxLDL positive MPs in groups between baseline collection and pretreatment stage.

The analysis of samples from pretreatment and posttreatment stages revealed that the CPos group had significantly increased numbers of MPs positive for* M. pneumoniae* (*P* = 0.02), as shown in Table 4; however, no significant differences in MPs positive for oxLDL were observed (*P* = 0.57) (Table 5). CNeg group did not exhibit a significant difference in any of the MPs types between pretreatment and posttreatment stages (Tables 4 and 5). When the groups were compared to each other, by means of Δ*T*, it was found that the numbers of MPs positive for* M. pneumoniae *were significantly higher in CPos group compared to animals in CNeg group (*P* = 0.002), while there was no difference regarding MPs positive for oxLDL antigens (*P* = 0.37) as shown in Tables 4 and 5 and Figure 4.

The treatment of animals with PTCTS reduced the number of MPs positive for oxLDL and* M. pneumoniae *antigens (Tables 4 and 5 and Figure 4) when the pretreatment and posttreatment phases were compared (*P* = 0.04 and *P* = 0.01, resp.), while the evaluation of Δ*T* showed that the treatment had a clear effect in reducing such MPs compared to the CPos group (*M. pneumoniae*, *P* < 0.001; oxLDL, *P* = 0.06).

The PTC treatment was not able to reduce the number of MPs positive for oxLDL antigens (*P* = 0.28) and showed only a tendency to remove MPs positive for* M. pneumoniae* antigens (*P* = 0.06). The comparison of Δ*T* showed that the treatment of animals with PTC had no significant effect on both MPs positive for* M. pneumoniae *(*P* > 0.05, Dunn test) and those positive for oxLDL (*P* = 0.11) antigens (Tables 4 and 5).

In comparing pretreatment versus posttreatment sera, TS group could not significantly reduce the number of MPs positive for oxLDL (*P* = 0.07) or* M. pneumoniae* (*P* = 0.13) antigens. When comparing the Δ*T* differences to CPos and PTCTS groups, TS group showed no differences in the count of both types of MPs (*P* = 0.11,* M. pneumoniae*,* t*-test Bonferroni; oxLDL, *P* > 0.05, Dunn test) (Tables 4 and 5).

### 3.4. Histological Analysis of the Organs

**Figure 5:**
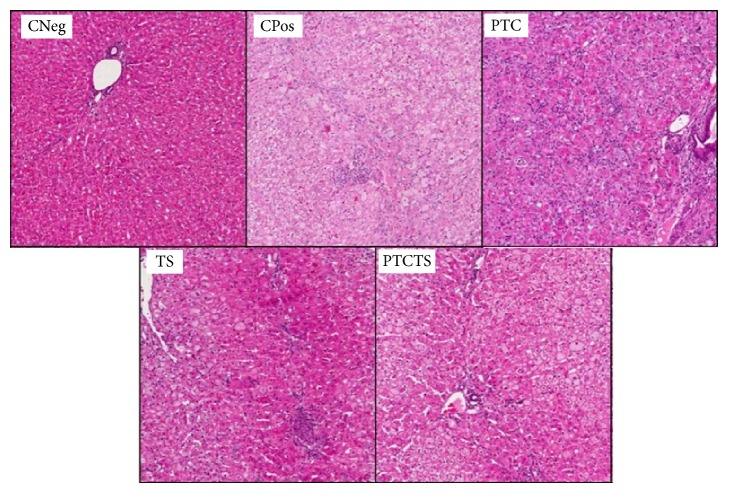
Representative images of histological analysis (H&E staining) of livers of rabbits from each group. Negative control (CNeg), positive control (CPos), and animals treated with PTC, TS, or PTCTS. Magnification, ×10.

**Figure 6:**
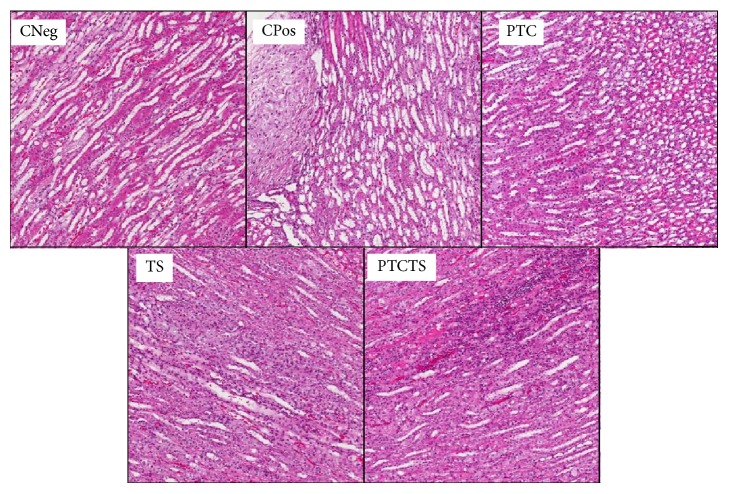
Representative images of histological analysis (H&E staining) of kidneys of rabbits from each group. Negative control (CNeg), positive control (CPos), and animals treated with PTC, TS, or PTCTS. Magnification, ×10.

**Figure 7:**
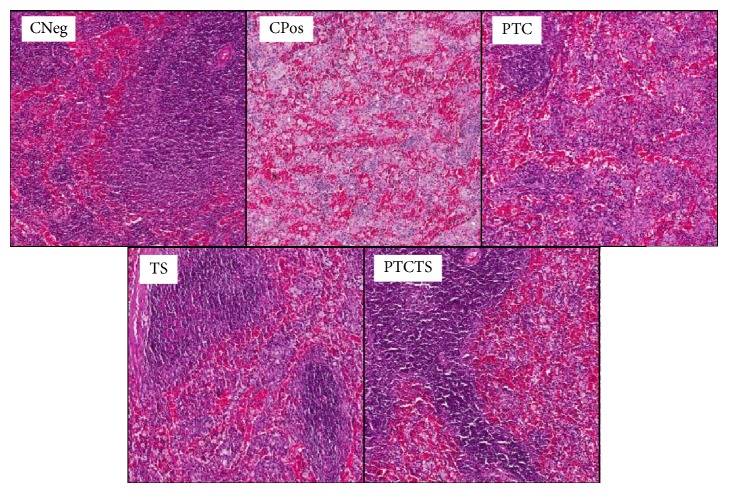
Representative images of histological analysis (H&E staining) of spleen of rabbits from each group. Negative control (CNeg), positive control (CPos), and animals treated with PTC, TS, or PTCTS. Magnification, ×10.

Histological analysis of liver, kidneys, and spleen of animals from CNeg group showed normal size (Table 6) and intact structures, as well as the absence of macroscopic lesions (Figure 5). The liver histology analysis of animals from CNeg group showed intact hepatocytes, space-door, arteries, ducts, and free septa, as well as absence of inflammation, pathological changes, or cell death (Figure 5). The kidneys had intact glomeruli and tubules and no bleeding, lymphocyte infiltration, or cell change (Figure 6). The spleen also showed full structure preserved red and white pulps and no infiltration of macrophages and/or fat, with no alterations or cell death (Figure 7).

CPos group showed organs with altered aspects, with significantly increased size of the liver compared to CNeg group (*P* = 0.02), and intense infiltration of lymphocytes, foam cells, and the presence of fat. The kidneys of animals from CPos group showed lymphocyte infiltration, the presence of fat, and typical cell change of fibrotic process, while the spleen contained xanthomatous macrophage and fat infiltration, besides presenting diffusion of red and white pulps.

The group treated with PTCTS showed livers with vacuolated hepatocytes, lymphocyte infiltration, and discrete presence of foam cells and fat to a lesser extent when compared to CPos. The kidneys of these animals contained discrete fat infiltration and mild bleeding, with intense lymphocytic infiltrate in the renal tubules, while the glomeruli showed no changes, similar to CNeg. In the spleen of animals from this group, discrete infiltration of foam cells was noted, with white and red pulps intact when compared to CNeg and CPos.

In animals from the PTC group, liver presented with lower presence of fat and xanthomatous macrophages but with intense lymphocytic infiltrate. A similar effect on the kidneys was noted, while spleen contained a moderate infiltration of foam cells and dispersion of red and white pulps.

Animals treated with TS showed lymphocytic and foam cells with moderate infiltration in the liver, kidneys, and spleen; the latter showed moderate dispersion of red and white pulps.

## 4. Discussion

Atherosclerosis is the consequence of the accumulation of monocytes and macrophages that invade the subendothelial space attracted by the presence of oxLDL. The phagocytosis of oxLDL particles by macrophages gives rise to xanthomatous cells, the base of the formation of atheroma [18].

Previous studies from our group showed that antigens and forms compatible with the bacteria* M. pneumoniae *and* Chlamydophila pneumoniae *were observed in atherosclerotic plaques and were even presenting larger amounts in ruptured plaques, which are responsible for acute myocardial infarction [15]. The presence of MPs in intimate contact with the bacteria was also described [19], as well as in circulating blood of infarcted patients [20, 21].

The present study evaluated whether MPs containing* M. pneumoniae* and oxLDL antigens are present in circulating blood from rabbits fed high cholesterol diet for 12 weeks and whether these MPs could be playing a role in the progression of the disease.

We also tested the impact of MP removal by the treatment of animals with PTCTS, a drug that was developed by H&S Science and Biotechnology, on the progression of atherosclerosis. We also assessed the effect of the individual components of PTCTS, PTC and TS, on the numbers of circulating MPs and the progression of atherosclerosis in rabbits. The cytotoxic effects of these compounds were also evaluated in key target organs (liver, kidneys, and spleen).

Before the start of the treatments with the compounds, it was possible to note that, as expected, the hypercholesterolemic diet increased serum levels of total cholesterol and its fractions in animals that were exposed to the diet in comparison to baseline.

In addition, we noted that there was an increase in serum MPs positive for* M. pneumoniae* and oxLDL antigens, even in CPos, which already had high baseline levels of MPs, indicating that* Mycoplasmas* and* Mycoplasma*-derived MPs may play a role in the development of atherosclerotic disease.

The treatment of animals with PTC did not show any lipid lowering properties when pretreatment and posttreatment stages were compared or in comparison to CPos and PTCTS groups. However, PTC could reduce the number of MPs positive for* M. pneumoniae *antigens, although not significantly, when we compared between pretreatment and posttreatment stages. Furthermore, PTC did not affect the number of MPs positive for oxLDL in comparison to animals from CPos and PTCTS groups.

In addition, the PTC treatment did not show a significant antiatherosclerotic effect since it was not capable of reducing plaque area in the ascending aorta, location known to be more affected by atherosclerotic disease, together with the aortic arch, due to high pressure blood and therefore more likely to exhibit endothelial injury [22]. However, the analysis of abdominal aorta showed a lesser plaque formation, although this was not statistically significant. This could be explained by the lower blood pressure and endothelial damage in this vascular territory as well as being the closest region to the site of uptake of organic particles, making thus this anatomical site the one showing the most visible effect of PTC.

Importantly, PTC treatment also showed important differences in the structure of the aorta with larger values of the external and internal perimeters compared to CPos, indicating that the vessel diameter in this group of the animals was greater. Such data associated with the potential and actual lumen values larger than the animals from CPos group, even with similar plaque area values, are indicative of positive remodeling, a phenomenon described by Glasgov et al. [23], in 1987, as compensatory enlargement of the coronary arteries to accommodate large quantities of atheromatous plaque, thus permitting the maintenance of blood flow.

The treatment of animals with TS significantly reduced serum HDL levels, although TG, n-HDL cholesterol, and total cholesterol levels remained high, which could be considered as a step towards an antilipidemic action. However, given alone, TS did not show any antiatherosclerotic effects in any of the analyzed portions of the aorta.

A negative correlation was previously observed between sialic acid levels of LDL and the capacity of these lipoprotein particles to cause intracellular accumulation of cholesterol [24]. Compared to native LDL, desialylated LDL has a higher capacity to bind to asialoglycoprotein receptor, a high capacity C-type lectin receptor, mainly expressed on hepatocytes. The interaction of desialylated LDL particles with this scavenger receptor leads to their uptake by the cell and hence to their intracellular accumulation due to reduced degradation [25]. The removal of sialic acid from LDL by neuraminidase was also found to enhance the atherogenic potential of LDL particles by increasing their capacity to accumulate intracellular cholesterol content in smooth muscle cells and macrophages [26]. Work by Tertov et al. [27] has later shown that human serum contained active TS and that the treatment of native (sialylated) LDL particles with TS caused desialylation of 60% of the LDL particles. When smooth muscle cells from healthy human aortic intima were exposed to TS-treated LDL particles, the intracellular cholesterol content significantly increased compared to cells treated with native LDL, indicating a proatherogenic potential for TS through its capacity to modify LDL and increase its intracellular uptake [27]. In our hands, and at the concentration used [1.62 × 10^−7^ mg/mL, 100 *μ*L/day], TS treatment was associated with a small antilipidemic effect and did not cause any further worsening of atherosclerosis in cholesterol-fed animals. Although an effect of the administered TS on circulating LDL particles cannot be completely ruled out, the daily dose of TS administered to rabbits in our study is very low compared to the concentration of TS which was shown to cause a modification of LDL particles [27]. Therefore, one can anticipate that, at the concentration used in our formulation, TS may not cause significant modifications of LDL particles and thus an important increase in the proportion of desialylated LDL; however, further investigation is warranted for confirmation.

Furthermore, the administration of TS alone showed no ability to remove MPs positive for* M. pneumoniae *or oxLDL antigens in circulating blood. This could be, partly, explained by the instability of TS in the acid pH of the stomach after oral administration.

We found here, however, that the combination of PTC and TS, PTCTS, was effective at several levels in atherosclerotic rabbits such as in removing MPs positive for oxLDL and* M. pneumoniae *antigens in the serum of rabbits after treatment and in lowing lipid levels, two factors of paramount importance in the development of antiatherogenic and anti-inflammatory response.

Furthermore, PTCTS demonstrated positive effects on the structure of the aorta by inducing changes in the vessel size as compared to negative and positive controls. These differences are again indicative of positive remodeling such as the one observed in the PTC group.

Even with no effects over the composition of the plaque or its vulnerability, the treatment of rabbits with PTCTS could reduce, although not significantly, the amount of plaque present in the abdominal portion of the aorta, suggesting a more effective antiatherosclerotic effect compared to PTC alone. The effects of treatment of rabbits with TS were different from those of the treatment with PTC; however, the effects of PTCTS presented characteristics from both components, indicating that the combination of the two components together is complementary.

The initiation of this study was based on previous work from our group, in which the PTCTS was administered intramuscularly to atherosclerotic rabbits and could remove almost completely plaque area in both the thoracic and abdominal portions or the aorta, as well as at the ascending aorta, while reducing the serum total cholesterol and its fractions and bringing them back to baseline levels [28]. However, the oral administration of the same compound did not produce the exact same results.

It is known that oral administration of medications has many advantages, such as the fact that it is a safe and practical way that allows self-administration as well as being more economical; however, it also has some disadvantages that should be taken into consideration. For instance, the absorption can range from moderate to low as well as high likelihood of partial loss of the drug by inactivation of the active ingredients due to the action of gastric enzymes and acid pH, while intramuscular route of administration is rapid and has systemic absorption.

The difference in the results with respect to antilipidemic and antiatherosclerotic actions of PTCTS may be due to either lesser plasma bioavailability with oral administration because of incomplete absorption or loss of some of the activity caused by the action of gastric enzymes.

Therefore, it is warranted to devise future pharmacokinetic studies to adjust the dose, treatment time, and delivery vehicle such that the compound holds its integrity even in the low pH environment of the stomach. We are currently testing the application of the compound in a stabilizing gel, which may eventually be included in gelatin capsules, allowing for a higher concentration of the drug to reach the bloodstream and target organs.

In conclusion, we have observed here that PTCTS components have different effects when administered alone. PTC treatment caused positive remodeling of the vessel, while the TS had lipid lowering effects with reduced TC in addition to the elimination of MPs positive for* M. pneumoniae* antigens.

When combined, the PTC and TS complemented the action of each other, allowing PTCTS to achieve more comprehensive actions and leading to a better therapeutic result, by improving lipid and liver profiles, the elimination of MPs, and causing positive vascular remodeling.

It is clearly warranted that, in order to achieve optimal results against atherosclerosis comparable to subcutaneously injected PTCTS, it is important to optimise the dosage, formulation, and the period of treatment for oral use of PTCTS.

Altogether, the findings reported in the present study reinforce the infectious theory of the pathogenesis of atherosclerosis and underscore the potential key role of circulating MPs originating from such microorganisms in the development of the disease. Therefore, the removal of these MPs from blood could be a new therapeutic approach in the treatment of atherosclerosis.

## Figures and Tables

**Table 1:** Circulating levels of total cholesterol, HDL cholesterol, non-HDL (n-HDL) cholesterol, and triglycerides in serum from control and treated groups at basal, pretreatment, and posttreatment phases. The difference between pretreatment and posttreatment stages (Δ*T*) is represented. Data are expressed as mean ± SD. Comparison between groups was performed using a paired *t*-test.

	Basal	Pretreatment	Posttreatment	Δ*T*	Basal × pre	Pre × post
					*P*	*P*
	Total cholesterol (mg/dL)					
CNeg	100 ± 85	88 ± 43	84 ± 55	−4 ± 17	0.32	0.58
CPos	65 ± 39	979 ± 274	1418 ± 383	439 ± 345	<0.001	0.03
PTC	74 ± 51	1183 ± 318	1534 ± 426	352 ± 586	<0.001	0.2
TS	74 ± 36	971 ± 145	960 ± 376	−9 ± 336	<0.001	0.95
PTCTS	89 ± 56	968 ± 293	1137 ± 273	169 ± 120	<0.001	0.02

	HDL (mg/dL)					
CNeg	37 ± 19	42 ± 17	32 ± 20	−10 ± 18	0.17	0.22
CPos	32 ± 17	506 ± 279	445 ± 131	−61 ± 193	0.004	0.47
PTC	32 ± 18	531 ± 91	487 ± 125	−44 ± 123	<0.001	0.42
TS	42 ± 14	615 ± 189	344 ± 137	−225 ± 205	0.001	0.03
PTCTS	39 ± 20	573 ± 158	442 ± 98	−131 ± 140	<0.001	0.07

	n-HDL (mg/dL)					
CNeg	63 ± 75	46 ± 31	52 ± 37	6 ± 14	0.26	0.33
CPos	33 ± 25	473 ± 193	973 ± 385	500 ± 256	0.002	0.005
PTC	42 ± 34	652 ± 292	1047 ± 375	395 ± 502	0.002	0.11
TS	32 ± 24	357 ± 206	616 ± 333	216 ± 246	0.01	0.08
PTCTS	50 ± 37	395 ± 395	696 ± 197	300 ± 229	0.01	0.02

	Triglycerides (mg/dL)					
CNeg	83 ± 27	56 ± 26	203 ± 335	147 ± 349	0.1	0.44
CPos	83 ± 59	134 ± 50	377 ± 201	244 ± 210	0.1	0.04
PTC	77 ± 27	205 ± 115	600 ± 556	396 ± 562	0.02	0.14
TS	51 ± 18	94 ± 44	344 ± 322	208 ± 277	0.02	0.12
PTCTS	69 ± 27	119 ± 91	386 ± 225	267 ± 139	0.35	0.005

**Table 2:** Plaque measurements of the ascending aorta from animals in each group after H&E staining. Data are expressed as mean ± SD.

	PE (mm)	PI (mm)	LP (mm^2^)	LR (mm^2^)	AP (mm^2^)	AM (mm^2^)	% Obs
CNeg	15.1 ± 1.3	12.3 ± 1.5	6.8 ± 1.6	6.8 ± 1.6	0 ± 0	5.9 ± 0.8	0 ± 0
CPos	15.1 ± 1.1	12.5 ± 1	5.9 ± 1.8	4.3 ± 1.7	2.1 ± 1.6	5.7 ± 0.4	28.6 ± 17.6
PTC	17.2 ± 1.7	14.7 ± 1.6	9.9 ± 3.1	7.3 ± 1.7	3 ± 2.6	6.7 ± 1.9	24.3 ± 11.7
TS	15.9 ± 0.6	13.2 ± 0.6	7.5 ± 2.5	5.9 ± 3	1.7 ± 1.7	6.3 ± 1.9	24.4 ± 17.7
PTCTS	17.1 ± 1.1	14.1 ± 1.3	9.7 ± 2.3	7.4 ± 2.8	2.3 ± 1.5	7.3 ± 1.1	24.5 ± 16.9

PE: external elastic lamina perimeter; PI: internal elastic lamina perimeter; AM: area of the middle layer; LP: potential lumen of the vessel, vessel lumen without the plaque; LR: real lumen of the vessel, vessel lumen with the plaque; AP: the area of atheromatous plaque; % Obs: percentage of vessel obstruction.

**Table 3:** Plaque measurements of thoracic and abdominal aorta from animals in each group after H&E staining. Data are expressed as mean ± SD.

	Thoracic portion			Abdominal portion		
	Total length (Et, mm)	Plaque length (Ep, mm)	Plaque area (AP, mm^2^)	Total length (Et, mm)	Plaque length (Ep, mm)	Plaque area (AP, mm^2^)
CNeg	10 ± 0.4	0.5 ± 1.2	0.03 ± 0.7	7.4 ± 2.0	0.1 ± 0.3	0.01 ± 0.02
CPos	9.7 ± 1.6	4.7 ± 3.6	0.8 ± 0.9	7.8 ± 2.4	4.1 ± 2.5	0.7 ± 0.8
PTC	10.6 ± 3.6	6.5 ± 3.7	1.4 ± 1.2	7.7 ± 2.8	2.6 ± 2.2	0.4 ± 0.4
TS	8.9 ± 2.2	4.4 ± 2.7	0.6 ± 0.4	8.9 ± 0.8	5 ± 3.6	1.1 ± 0.9
PTCTS	8.7 ± 1.8	3.9 ± 2.8	0.7 ± 0.4	7.6 ± 1.4	3.1 ± 2.5	0.3 ± 0.3

**Table 4:** Number of circulating MPs associated with *M. pneumoniae* antigens in animals from each group. Data are expressed as mean ± SD.

	*Mycoplasma pneumoniae*				Basal × pre	Pre × post
	Basal	Pretreatment	Posttreatment	Δ*T*	*P*	*P*
CNeg	45 ± 16	71 ± 25	49 ± 17	−22 ± 29	0.02	0.12
CPos	209 ± 104	286 ± 106	437 ± 199	152 ± 112	0.001	0.02
PTC	62 ± 42	107 ± 43	70 ± 14	−36 ± 49	0.08	0.06
TS	369 ± 243	286 ± 158	361 ± 135	75 ± 87	0.31	0.13
PTCTS	102 ± 59	296 ± 184	159 ± 103	−137 ± 88	0.02	0.01

**Table 5:** Number of circulating MPs associated with oxLDL in animals from each group. Data are expressed as mean ± SD.

	oxLDL				Basal × pre	Pre × post
	Basal	Pretreatment	Posttreatment	Δ*T*	*P*	*P*
CNeg	335.3 ± 250.1	386.7 ± 232.4	301 ± 203.3	−85.7 ± 277.2	0.13	0.48
CPos	185.3 ± 112.6	305.8 ± 239.6	407.8 ± 254.6	102 ± 406.6	0.09	0.57
PTC	206.3 ± 127.4	314.7 ± 76.1	223 ± 154.1	−91.7 ± 187.1	0.07	0.28
TS	267.4 ± 171.1	261 ± 190.3	311.6 ± 169.44	50.6 ± 45.9	0.26	0.07
PTCTS	236.7 ± 120.7	350 ± 86.3	204.5 ± 57.9	−146 ± 126.6	0.05	0.04

**Table 6:** Weight of liver, kidneys, and spleen of animals in each group. Data are expressed as mean ± SD.

	Liver (g)	Kidneys (g)	Spleen (g)
CNeg	95.3 ± 20.5	22.5 ± 3.4	2.8 ± 0.7
CPos	131.4 ± 25.7	21.2 ± 6.2	3.9 ± 1.4
PTC	145.2 ± 12.5	23.9 ± 3.7	7 ± 3.4
TS	143.9 ± 24	26.8 ± 7.5	6.2 ± 2.2
PTCTS	146.8 ± 16.6	23.7 ± 2.1	5.7 ± 1.7
